# Minor hallucinations in Parkinson’s disease with probable rapid eye movement sleep behavior disorder

**DOI:** 10.3389/fnins.2023.1205439

**Published:** 2023-08-14

**Authors:** Yinyin Jiang, Jun Zhu, Yang Zhao, Dongfeng Li, Yaning Chen, Yaxi Wang, Xu Jiang, Bo Shen, Yang Pan, Jun Yan, Feng Han, Li Zhang

**Affiliations:** ^1^Department of Geriatric Neurology, Nanjing Brain Hospital Affiliated to Nanjing Medical University, Nanjing, China; ^2^School of Pharmacy, Nanjing Medical University, Nanjing, China; ^3^Institute of Brain Science, Nanjing Brain Hospital Affiliated to Nanjing Medical University, Nanjing, China; ^4^Institute of Neuropsychiatric Diseases, Nanjing Brain Hospital Affiliated to Nanjing Medical University, Nanjing, China

**Keywords:** Parkinson’s disease, REM sleep behavior disorder, RBDSQ, minor hallucination, presence hallucination

## Abstract

**Background:**

Rapid eye movement sleep behavior disorder (RBD) and minor hallucinations (MHs) are prevalent nonmotor symptoms in Parkinson’s disease (PD). The purpose of this study was to explore the association of MHs in PD patients with probable RBD (pRBD).

**Methods:**

This cross-sectional study included 291 patients diagnosed with PD. Patients who scored 6 or higher on the Rapid Eye Movement Behavior Disorder (RBD) Screening Questionnaire were defined as pRBD. A comprehensive evaluation was performed for all patients, including the collection of demographic information, clinical assessment, and MH features.

**Results:**

Among the 291 PD patients, 69 (23.7%) had pRBD. MHs were observed in 35 (50.7%) patients with pRBD, significantly higher than 29.7% in patients without RBD (*p* = 0.015). The main type of MHs in pRBD was presence hallucinations with variable content. Patients with pRBD and MHs tended to be older, had a longer disease duration, and were more likely to take levodopa or dopamine-receptor agonists. Besides, the pRBD with MHs group had higher scores on the Nonmotor Symptoms Questionnaire (NMS-Quest) and Hamilton Anxiety Scale (HAMA). Binary logistic regression analysis revealed that longer disease duration and higher NMS-Quest scores were associated with MHs in PD patients with pRBD.

**Conclusion:**

A high prevalence of MHs was observed in PD patients with pRBD. The main type of MHs in pRBD was presence hallucinations. MHs in PD with RBD are mainly associated with disease duration and severity of nonmotor symptoms. These findings provide new insights into the interaction between MHs and RBD.

## 1. Introduction

Parkinson’s disease (PD) is a common chronic neurodegenerative disorder characterized by motor symptoms (Bloem et al., 2021). However, patients with PD suffer greatly from various nonmotor symptoms, such as sleep disturbances, psychotic symptoms, and autonomic dysfunction (Schapira et al., 2017). Rapid eye movement sleep behavior disorder (RBD) is the most frequent sleep disorder in PD patients and is characterized by dream-enacting behavior and loss of normal muscle atonia during rapid eye movement (REM) sleep (Dauvilliers et al., 2018; Postuma et al., 2019). Previous studies have shown that PD patients with RBD may experience more severe autonomic and psychiatric symptoms (Sixel-Döring et al., 2011; Fujita et al., 2022), and PD with RBD may be a more aggressive subtype of the disease (Liu et al., 2021).

Minor hallucinations (MHs) and well-structured visual hallucinations (VHs) are the most common manifestations of psychotic symptoms in PD (Ffytche et al., 2017; Clegg et al., 2018). Previous studies have reported a significant association of RBD with VHs in PD patients (Onofrj et al., 2006; Wu et al., 2016). A 2-year prospective study reported that patients with PD and RBD have a higher risk of developing VHs than those without RBD (Sinforiani et al., 2008). The interactions between RBD and VHs have been extensively explored. MHs consist of presence hallucinations, passage hallucinations, and illusions, which usually precede the onset of VHs (Lenka et al., 2019). Several studies have revealed that RBD is an independent predictor of MHs in PD patients (Lenka et al., 2017; Zhong et al., 2021). However, few studies have investigated MHs in PD patients with RBD. The characteristics of MHs in PD with RBD remain unclear.

The aim of this study was to explore the association of MHs in PD patients with probable RBD (pRBD), especially the characteristic spectrum of MHs, and to investigate the potential risk factors for MHs in this population. These findings may offer new insights into the interaction between RBD and MHs in Parkinson’s disease.

## 2. Materials and methods

### 2.1. Patients

From April 2020 to October 2022, 384 patients with PD were consecutively recruited from the Department of Geriatrics at Nanjing Brain Hospital, affiliated with Nanjing Medical University. The diagnosis of PD was made by at least two specialists in movement disorder disease according to the United Kingdom Parkinson’s Disease Society Brain Bank criteria (Hughes et al., 1992). The exclusion criteria for this study were as follows: (1) history of major psychiatric diseases (or use of any antipsychotic medication) and focal brain injury (according to MRI evidence); (2) significant cognitive impairment, based on a Montreal Cognitive Assessment (MoCA) score ≤ 20 (Gill et al., 2008; Dalrymple-Alford et al., 2010); (3) Scored over 1 point on the MDS-UPDRS part 1 hallucination & psychosis item; (4) abnormal version or corrected vision, or (5) other serious medical diseases. We excluded six patients for cerebrovascular disease, cerebral atrophy, and malignant tumors; 35 for major psychiatric diseases or use of antipsychotic medication; 43 for significant cognitive impairment; eight for formed hallucinations or delusions; and one for glaucoma. Finally, a total of 291 patients were included in this cross-sectional study.

We applied a classification of probable RBD (pRBD) according to the REM Sleep Behavior Disorder Screening Questionnaire (RBDSQ) as used in several previous studies (Long et al., 2020; Ashraf-Ganjouei et al., 2021; Barasa et al., 2021; Pavelka et al., 2022). Assignment of pRBD to idiopathic PD individuals used the criterion RBDSQ score ≥ 6 to optimize the specificity and sensitivity for pRBD in line with the Oxford Discovery Study (Liu et al., 2021).

The presence of hallucinations was assessed based on the “Hallucinations and Psychosis” item of the Movement Disorder Society-Sponsored Revision of the Unified Parkinson’s Disease Rating Scale (MDS-UPDRS) Part 1. Response options were as follows: 0 = no hallucinations or psychotic behavior, 1 = minor hallucinations, 2 = formed hallucinations without loss of insight, 3 = formed hallucinations with loss of insight, or 4 = delusions (Goetz et al., 2008). Patients with a score of 1 and whose MHs appeared stable in the last month were included in the MH+ group.

This study was approved by the Ethics Committee of Nanjing Brain Hospital affiliated with Nanjing Medical University, and written informed consent was obtained from all participants.

### 2.2. Clinical assessment

Structured interviews were conducted using a standardized questionnaire to collect general information such as name, gender, age, body mass index (BMI), education level, age at PD onset, disease duration, use of antiparkinsonian drugs, and levodopa-equivalent daily dose (LEDD; Tomlinson et al., 2010). Based on our previous research, a more comprehensive questionnaire was applied to obtain the detailed characteristics of MHs (Zhong et al., 2021). Disease severity was rated using the Hoehn and Yahr stage (H-Y stage; Hoehn and Yahr, 1967). Motor symptoms were assessed by the Unified Parkinson’s Disease Rating Scale (UPDRS) part III score while nonmotor symptoms were measured by the Nonmotor Symptom Questionnaire (NMS-Quest; Fahn, 1987; Chaudhuri et al., 2006). Cognitive function was assessed by the Montreal Cognitive Assessment (MoCA). The Parkinson’s Disease Sleep Scale (PDSS) was used to measure sleep quality, and the Rapid Eye Movement Sleep Behavior Disorder Screening Questionnaire (RBDSQ) was used to further screen for probable RBD (Chaudhuri et al., 2002; Stiasny-Kolster et al., 2007). Besides, the 15th item (also the last) of the PDSS scale was separately calculated to evaluate daytime sleepiness symptoms. The item was the question “Have you unexpectedly fallen asleep during the day?,” and the score for this item ranged from 0 to 10 (10 represents excellent/never responses, and 0 illustrates the worst score). All patients completed the Hamilton Anxiety Rating Scale (HAMA) and the Hamilton Rating Scale for Depression (HAMD) to evaluate the severity of anxiety and depression, respectively (Hamilton, 1959; Williams, 1988). The quality of life of our patients was assessed using the 39-Item Parkinson’s Disease Questionnaire (PDQ-39; Jenkinson et al., 1997).

### 2.3. Statistical analysis

IBM SPSS 26.0 software was used for statistical analysis. Measurement data are expressed as the mean ± standard deviation (SD) or as number (N) or percentage (%). The Kolmogorov–Smirnov test was used to check the normality of the measurement data. A two-sample *t* test was used for normally distributed data, and the Mann–Whitney U test was used for nonnormally distributed data. Categorical variables are shown as percentages and were analyzed using a chi-square test. The MH (%), NMS-Quest, PDSS, MoCA, HAMA, HAMD, and PDQ-39 scores were compared by one-way analyses of covariance (ANCOVA) with adjustments for confounding factors, including age and disease duration. A logistic regression model was used to identify possible factors associated with hallucinations in patients with probable RBD. Univariate regression analysis was first performed to screen statistically significant variables. After excluding the effects of confounding factors, including age and disease duration, all significant variables were then entered into the multivariate regression model. Values of *p* < 0.05 were deemed statistically significant.

## 3. Results

### 3.1. Comparison of demographic information and clinical scale data between PD-pRBD and PD-nRBD groups

A total of 291 patients with PD were recruited for this study, including 69 (23.7%) PD-pRBD patients and 222 (76.3%) PD-nRBD patients. Among them, 101 (34.6%) patients experienced MHs, including 35 (50.7%) patients in the PD-pRBD group and 66 (29.7%) patients in the PD-nRBD group. As shown in Table 1, there were no differences in age, sex, BMI, education level, age of onset, H-Y stage, UPDRS-III scores, or LEDD between the two groups. However, the PD-pRBD group had a longer disease duration and a higher proportion of individuals that used dopamine receptor agonists than PD-nRBD, while other drug types did not differ between the two groups. The differences between the PD-pRBD and PD-nRBD groups were not significant regarding PDSS-item15, MoCA, HAMD, and PDQ-39 scores, while the differences in NMS-Quest, PDSS, and HAMA scores were significant. Besides, there was a significantly higher proportion of patients in pRBD group who had MH than those without RBD (*p* = 0.015).

**Table 1:** Comparison of demographic information and clinical scale data between PD-pRBD and PD-nRBD groups.

	All patients	PD-pRBD	PD-nRBD	*P*
	*N* = 291	*N* = 69	*N* = 222	
Age (years)	66.40 ± 9.34	66.17 ± 9.17	66.48 ± 9.41	0.687^a^
Gender (%)	152 (52.2%)	41 (59.4%)	111 (50.0%)	0.171^c^
BMI	23.65 ± 3.47	23.62 ± 2.88	23.65 ± 3.64	0.942^a^
Education (%)				0.523^c^
Illiteracy	45 (15.5%)	14 (20.3%)	31 (14.0%)	
Primary school	40 (13.7%)	7 (10.1%)	33 (14.9%)	
Middle school	149 (51.2%)	35 (50.7%)	114 (51.4%)	
College or above	57 (19.6%)	13 (18.8%)	44 (19.8%)	
Age of onset	60.74 ± 10.38	59.35 ± 9.70	61.17 ± 10.57	0.204^b^
Disease duration (years)	5.76 ± 4.51	7.07 ± 5.44	5.35 ± 4.11	**0.034**^a^
H-Y stage	2.46 ± 0.70	2.50 ± 0.58	2.44 ± 0.73	0.463^a^
UPDRS-III off	31.53 ± 15.83	31.87 ± 13.72	31.42 ± 16.46	0.620^a^
PD treatment				
Levodopa	216 (74.2%)	55 (79.7%)	161 (72.5%)	0.233^c^
Dopamine agonists	161 (55.3%)	46 (66.7%)	115 (51.8%)	**0.030**^c^
MAO-B inhibitors	36 (12.4%)	11 (15.9%)	25 (11.3%)	0.302^c^
COMT inhibitors	24 (8.2%)	8 (11.6%)	16 (7.2%)	0.247^c^
Amantadine	61 (21.0%)	17 (24.6%)	44 (19.8%)	0.391^c^
Benzhexol	24 (8.2%)	6 (8.7%)	18 (8.1%)	0.877^c^
LEDD (mg)	575.53 ± 482.84	634.68 ± 449.37	571.52 ± 492.43	0.119^a^

MH (%)	101 (34.6%)	35 (50.7%)	66 (29.7%)	**0.015**^c^
NMS-Quest	12.38 ± 5.38	16.16 ± 4.27	11.21 ± 5.16	**<0.001**^b^
PDSS	107.07 ± 28.12	98.12 ± 29.34	109.85 ± 27.20	**0.020**^a^
PDSS-item15	6.65 ± 3.46	6.18 ± 3.48	6.78 ± 3.45	0.192^b^
MoCA	25.36 ± 2.88	24.38 ± 2.74	25.62 ± 2.51	0.458^a^
HAMA	6.99 ± 4.68	8.64 ± 4.80	6.48 ± 4.53	**0.002**^a^
HAMD	8.31 ± 5.09	9.35 ± 4.72	7.98 ± 5.12	0.157^a^
PDQ-39	45.76 ± 27.28	52.23 ± 25.73	43.75 ± 27.49	0.187^a^

Variables are expressed as mean ± standard deviation (SD) or number (percentage). ^a^Student’s *t*-test, ^b^Mann–Whitney U-test, and ^c^Chi-square test. The bold values symbolized statistically significant differences (*p* < 0.05). The *p* value of MH% and clinical scales were adjusted for age and disease duration. PD, Parkinson’s disease; pRBD, probable rapid eye movement sleep behavior disorder; BMI, body mass index; H-Y stage, Hoehn-Yahr stage; UPDRS III, Unified Parkinson’s Disease Rating Scale Part III; LEDD, Levodopa equivalent daily dose; MH, minor hallucination; NMS-Quest, Non-motor symptoms questionnaire; PDSS, Parkinson’s Disease Sleep Scale; PDSS-item15, “Have you unexpectedly fallen asleep during the day?”; MoCA, montreal cognitive assessment; HAMA, Hamilton anxiety rating scale; HAMD, Hamilton depression rating scale; PDQ39, Parkinson’s disease questionnaire-39.

### 3.2. Comparison of demographic information and clinical scale data in PD-pRBD patients based on the presence/absence of MHs

Table 2 shows the comparison between the pRBD-MH and pRBD-NH groups. Among all 69 PD-pRBD patients, 35 (50.7%) experienced MHs. Patients in the PD-pRBD group with MHs were older and had a longer disease duration. There were significant differences in the use proportion of levodopa and dopamine agonists between groups. Levodopa was used by 32 (91.4%) pRBD-MH patients and 23 (67.6%) pRBD-NH patients. Dopamine agonists were used in 28 (80%) pRBD-MH patients and 18 (52.9%) pRBD-NH patients. No significant differences were found in sex, BMI, education level, age of onset, H-Y stage, UPDRS-III score, or LEDD. We observed that NMS-Quest and HAMA scores were significantly higher in pRBD-MH patients. There were no significant group differences in PDSS, PDSS-item15, MoCA, HAMD, and PDQ-39 scores.

**Table 2:** Comparison of demographic information and clinical scale data in PD-pRBD patients based on the presence/absence of MHs.

	PD-pRBD			*P*
	All	MH	NH	
Number	69 (100%)	35 (50.7%)	34 (49.3%)	
Age (years)	66.17 ± 9.17	68.97 ± 8.20	63.29 ± 9.34	**0.009**^a^
Gender (%)	41 (59.4%)	17 (48.6%)	24 (70.6%)	0.063^c^
BMI	23.62 ± 2.88	23.39 ± 2.96	23.85 ± 2.82	0.511^a^
Education (%)				0.682^c^
Illiteracy	14 (20.3%)	7 (20.0%)	7 (20.6%)	
Primary school	7 (10.1%)	3 (8.6%)	4 (11.8%)	
Middle school	21 (30.4%)	9 (25.7%)	12 (35.3%)	
College or above	27 (39.1%)	16 (45.7%)	11 (32.3%)	
Age of onset	59.35 ± 9.70	60.57 ± 9.78	58.03 ± 9.66	0.281^b^
Disease duration (years)	7.07 ± 5.44	8.66 ± 5.57	5.44 ± 4.85	**0.008**^a^
H-Y stage	2.50 ± 0.58	2.57 ± 0.56	2.43 ± 0.60	0.170^a^
UPDRS-III off	31.87 ± 13.72	33.09 ± 14.21	30.62 ± 13.29	0.494^a^
PD treatment				
Levodopa	55 (79.7%)	32 (91.4%)	23 (67.6%)	**0.014**^c^
Dopamine agonists	46 (66.7%)	28 (80.0%)	18 (52.9%)	**0.017**^c^
MAO-B inhibitors	11 (15.9%)	5 (14.3%)	6 (17.6%)	0.703^c^
COMT inhibitors	8 (11.6%)	4 (11.4%)	4 (11.8%)	0.963^c^
Amantadine	17 (24.6%)	8 (22.8%)	9 (26.5%)	0.728^c^
Benzhexol	6 (8.7%)	4 (11.4%)	2 (5.9%)	0.414^c^
LEDD (mg)	634.68 ± 449.37	715.29 ± 395.84	551.69 ± 490.58	0.112^a^

NMS-Quest	16.16 ± 4.27	18.20 ± 3.72	14.06 ± 3.79	**<0.001**^b^
PDSS	98.12 ± 29.34	91.50 ± 29.02	104.941 ± 28.51	0.071^a^
PDSS-item15	6.18 ± 3.48	5.74 ± 3.97	6.64 ± 2.90	0.322^b^
MoCA	24.38 ± 2.74	23.71 ± 2.49	24.46 ± 2.27	0.499^a^
HAMA	8.64 ± 4.80	9.97 ± 4.43	7.26 ± 4.85	**0.026**^a^
HAMD	9.35 ± 4.72	10.54 ± 4.98	8.21 ± 4.15	0.063^a^
PDQ-39	52.23 ± 25.73	59.26 ± 25.22	45.00 ± 24.54	0.064^b^

Variables are expressed as mean ± standard deviation (SD) or number (percentage). ^a^Student’s *t*-test, ^b^Mann–Whitney U-test, and ^c^Chi-square test. The bold values symbolized statistically significant differences (*p* < 0.05). The *p* value of clinical scales was adjusted for age and disease duration. PD, Parkinson’s disease; pRBD, Probable rapid eye movement sleep behavior disorder; MH, Minor hallucination; NH, No hallucinations; BMI, Body mass index; H-Y stage, Hoehn-Yahr stage; UPDRS III, Unified Parkinson’s disease rating scale part III; LEDD, Levodopa equivalent daily dose; MH, minor hallucination; NMS-Quest, Non-motor symptoms questionnaire; PDSS, Parkinson’s disease sleep scale; PDSS-item15, “Have you unexpectedly fallen asleep during the day?”; MoCA, Montreal cognitive assessment; HAMA, Hamilton anxiety rating scale; HAMD, Hamilton depression rating scale; and PDQ39, Parkinson’s disease questionnaire-39.

### 3.3. Characteristics of minor hallucinations in the PD-pRBD and PD-nRBD groups

**Figure 1:**
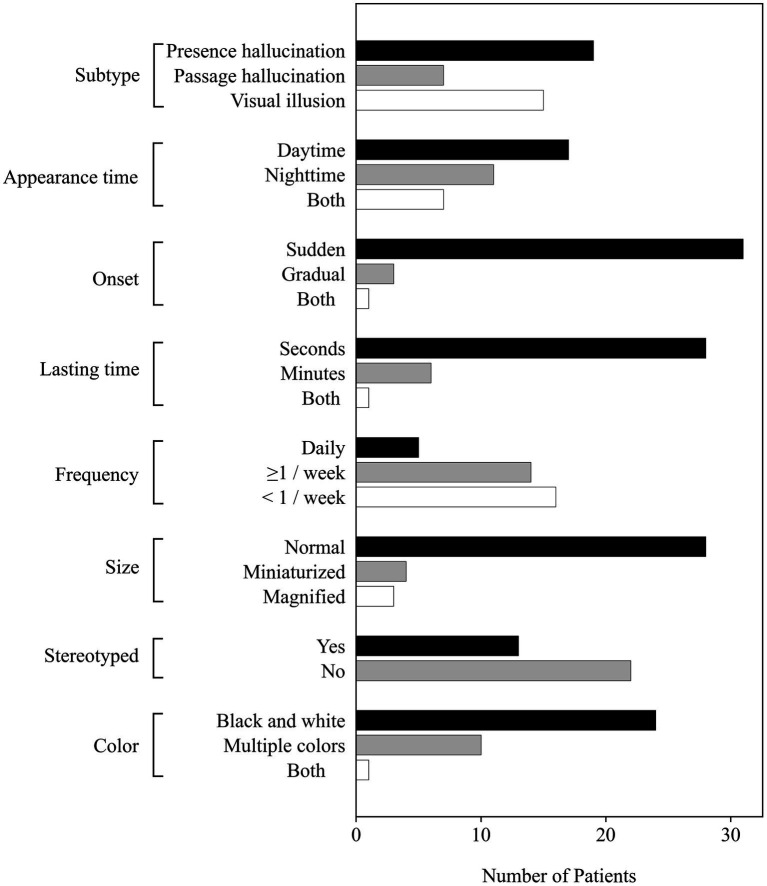
Characteristics of minor hallucinations in PD patients with probable RBD. Of the 69 PD patients with probable RBD recruited in our study, 35 (50.7%) experienced MHs. Among them, 19 (54.3%) had presence hallucinations, 7 (20.0%) had passage hallucinations, and 15 (42.8%) had visual illusions. In terms of appearance time of MHs, 17 (48.6%) patients appeared at daytime, 11 (31.4%) patients appeared at nighttime, and 7 (20%) patients appeared at both times. MHs of 31 (88.6%) patients appeared suddenly, and 3 (8.6%) patients appeared gradually. MHs of 28 (80%) patients lasted seconds, and six patients lasted minutes. Only five patients had MHs daily, 14 patients had MHs 1–6 times per week, and 16 patients had MHs less than one time per week. As for the size of MHs, 28 (80%) patients had normal size images, 4 (11.4%) patients had miniaturized images, and 3 (8.6%) patients had magnified images. Hallucinatory images were stereotyped in 13 (37.1%) patients and variable in 22 (62.9%). The color of MHs was black and white in 24 (68.6%) patients and colorful in 10 (28.8%) patients.

Table 3 summarizes the characteristics of MHs. In this study, 101 PD patients experienced MHs, including 35 (50.7%) patients in the PD-pRBD group and 66 (29.7%) patients in PD-nRBD. There were significant differences in the types of MHs between groups. Presence hallucinations (54.3%) were more common in the PD-pRBD group (*p* = 0.007), while in PD-nRBD, passage hallucinations (47.7%) were more frequent (*p* = 0.005). The prevalence of visual hallucinations was 42.8% in the PD-pRBD group and 35.4% in PD-nRBD. Additionally, compared with the stereotyped MHs in the PD-nRBD group, MHs in PD-pRBD patients were more variable (*p* = 0.035). MHs could appear at any time of the day and tended to appear suddenly and last for only a few seconds in the two groups. The frequency of MHs ranged from daily to less than once a week, but daily episodes were less common in the two groups. Also, the hallucinated images were both normal and monochromatic in the two groups. The characteristics of MHs in the PD-pRBD group are shown in Figure 1.

**Table 3:** Characteristics of minor hallucinations in the PD-pRBD and PD-nRBD groups.

	All MH	pRBD-MH	nRBD-MH	*P*
	*N* = 101	*N* = 35	*N* = 66	
Subtype				
Presence hallucination	37 (36.6%)	19 (54.3%)	18 (27.7%)	**0.007**
Passage hallucination	39 (38.6%)	7 (20.0%)	32 (47.7%)	**0.005**
Visual illusion	38 (37.6%)	15 (42.8%)	23 (35.4%)	0.429
Appearance time				0.290
Daytime	52 (51.5%)	17 (48.6%)	35 (53%)	
Nighttime	36 (35.6%)	11 (31.4%)	25 (37.9%)	
Both	13 (12.9%)	7 (20%)	6 (9.1%)	
Onset				0.219
Sudden	86 (85.1%)	31 (88.6%)	55 (83.3%)	
Gradual	14 (13.9%)	3 (8.6%)	11 (16.7%)	
Both	1 (1.0%)	1 (2.8%)	0 (0%)	
Lasting time				0.862
Seconds	83 (82.2%)	28 (80%)	55 (83.3%)	
Minutes	16 (15.8%)	6 (17.1%)	10 (15.2%)	
Both	2 (2.0%)	1 (2.8%)	1 (1.5%)	
Frequency				0.461
Daily	21 (20.8%)	5 (14.3%)	16 (24.2%)	
≥1/week	35 (34.6%)	14 (40.0%)	21 (31.8%)	
<1/week	45 (44.6%)	16 (45.7%)	29 (43.9%)	
Size				0.615
Normal	85 (84.2%)	28 (80%)	57 (86.4%)	
Miniaturized	8 (7.9%)	4 (11.4%)	4 (6.1%)	
Magnified	8 (7.9%)	3 (8.6%)	5 (7.5%)	
Stereotyped				**0.035**
Yes	52 (51.5%)	13 (37.1%)	39 (59.1%)	
No	49 (48.5%)	22 (62.9%)	27 (40.9%)	
Color				0.484
Black and white	76 (75.2%)	24 (68.6%)	52 (78.8%)	
Multiple colors	22 (21.9%)	10 (28.8%)	12 (18.2%)	
Both	3 (3.0%)	1 (2.8%)	2 (3.0%)	

Variables are expressed as number (percentage). The bold values symbolized statistically significant differences (*p* < 0.05). PD, Parkinson’s disease; pRBD, probable rapid eye movement sleep behavior disorder; and MH, minor hallucination.

### 3.4. Risk factors for MHs in PD patients with probable RBD

As shown in Table 4, the binary logistic regression analysis revealed that a longer disease duration [odds ratio (OR) 1.136, 95% confidence interval (CI) 1.012–1.274] was significantly associated with a greater risk of MHs in PD patients with probable RBD. Higher scores on the NMS-Quest (OR 1.276; 95% CI 1.076–1.514) may also be a risk factor. However, age and depression symptoms were not closely related to MHs in PD-pRBD.

**Table 4:** Binary logistic regression analysis for independent predictors of minor hallucinations in PD patients with probable RBD.

	OR	95%CI	*P*
Age	1.043	0.974–1.118	0.228
Disease duration	1.136	1.012–1.274	**0.030**
NMS-Quest	1.276	1.076–1.514	**0.005**
HAMA	1.081	0.945–1.236	0.257

The bold values symbolized statistically significant differences (*p* < 0.05). OR, odds ratio; CI, confidence interval; NMS-Quest, Non-motor symptoms questionnaire; and HAMA, Hamilton anxiety rating scale.

## 4. Discussion

In this cross-sectional study, MHs were detected in 34.6% of patients in our cohort. We found that the prevalence of minor hallucinations (MHs) in patients with pRBD was up to 50.7%, the main type of which was presence hallucinations. The association between self-reported RBD and MHs was explained by longer disease duration and more severe nonmotor symptoms. To the best of our knowledge, this is the first study to explore the associations of MHs in PD patients with RBD.

Numerous articles have proved significant associations between RBD and MHs (Forsaa et al., 2010; Pagonabarraga et al., 2016; Barrett et al., 2017; Omoto et al., 2021). Zhong et al. (2021) reported that patients with MHs have higher RBDSQ scores and RBD is a risk factor for MHs in PD patients. In our study, the prevalence of MHs in pRBD patients was up to 50.7%, significantly higher than 29.7% in nRBD. This finding also showed that RBD and MHs were related. Cholinergic dysfunction may account for this relation. The cholinergic magnocellular nuclei of the basal forebrain complex are affected by Lewy body pathology in Braak stage 3 (Braak et al., 2003). Notably, the basal forebrain complex has strong interconnections with the brainstem nuclei regulating sleep and atonia during REM. Therefore, RBD symptoms may signal cholinergic system degeneration (Nardone et al., 2017). Although the pathophysiology of MHs and VHs is still under exploration, they are thought to be related to the disruption of visual processing and cholinergic dysfunction (Collerton et al., 2005).

Furthermore, neuroimaging studies also provided evidence. The increased psychiatric disturbances in PD with RBD were considered due to damage to key limbic regions (Matzaras et al., 2022). The typical PD-associated psychosis (PDP) is a continuous process that begins with MHs and then evolves into major hallucinations with insight, followed by gradual loss of insight and eventually delusions (Ffytche et al., 2017). Although only a few articles have been published on imaging studies of MHs, several similar structural and functional characteristics between MHs and VHs have been found. Apart from gray matter atrophy in limbic and paralimbic regions in patients with VHs (Lenka et al., 2015), morphometric studies also reported reduced gray matter volume of posterior cingulate cortex and parahippocampal gyri in patients with MHs (Bejr-Kasem et al., 2019; Zhong et al., 2022). Considering that RBD represents a more aggressive PD subtype (Fereshtehnejad et al., 2015), we conjecture that MHs in this population may be associated with damage to related brain regions in the early stage.

Previous studies reported that visual illusion was the most common type of MHs in patients with PD (Barrett et al., 2017). However, we found that over half of PD patients with pRBD had presence hallucinations. Presence hallucination refers to the vivid perception of the presence of someone other than oneself, usually to the side or behind oneself, in the absence of evidence for this perception (Fénelon, 2008). Previous studies proved that presence hallucinations did not tend to be stereotypical in nature (Fénelon et al., 2011; Wood et al., 2015). This explains why MHs of RBD patients showed more variability in our study. Besides, evidence has emerged that three minor hallucinations are associated with different risk factors (Ffytche et al., 2017). Archibald et al. (2011) reported that RBD was an independent predictor of presence hallucinations. Given that presence hallucinations are non-visual perceptions, cortical lesions rather than disorders of primary sensory pathways probably attribute to its generation (Sanchez-Ramos et al., 1996). Bernasconi et al. (2021) team combined MR-compatible robotics and revealed the pathological cortical sensorimotor processes of presence hallucinations in PD. Considering both RBD and psychosis are predictors of Lewy body pathology, the association of VHs with a high density of Lewy bodies in the temporal lobe may provide a clue as to the cortical substrate of presence hallucinations in patients with RBD (Harding et al., 2002).

Although the mechanism by which different MHs develop into VHs may vary, several studies reported a close association between presence hallucinations and VHs (Morgante et al., 2012). Fénelon et al. (2011) observed that 1/3 of PD patients with unformed VHs also had presence hallucinations, and presence hallucination could predict well-structured hallucinations (odds ratio: 4.5). Therefore, we speculate that VHs in patients with RBD, later in the course of the disease, may probably develop from the initial presence hallucinations. It is necessary to further investigate the pathogenesis of MHs in PD patients with RBD, especially the evolution of presence hallucination, and explore the relationship of MHs with VHs.

In PD-pRBD patients with MHs, we observed a higher use proportion of dopamine agonists. Although previous studies reported that dopamine agonists establish some side effects, including hallucinations and other psychosis in PD (Stowe et al., 2008; Morgante et al., 2012), research on visual hallucinations suggests that medications are only a precipitating factor for hallucinations, not their cause (Ravina et al., 2007; Powell et al., 2020). Other factors such as age, disease duration, cognitive status, and sleep–wake cycle disturbances may also contribute to this (Sanchez-Ramos et al., 1996; Fénelon et al., 2000). Pagonabarraga reported that MHs can occur in drug-naïve patients and even before the onset of motor symptoms (Pagonabarraga et al., 2016). Also, we found that medications are not the independent risk factors for MHs.

Rapid eye movement sleep behavior disorder and MHs are two common nonmotor symptoms in Parkinson’s disease that impose a substantial burden on patients and their caregivers. Arnulf et al. (2000) found an association between VHs and REM sleep episodes during daytime and proposed that the images of VHs may be attributed to REM sleep episodes intruding into wakefulness. Although we observed MHs patients performed worse on somnolence scores (PDSS-item15), we did not find any significant differences between groups. This may be due to the MHs in our study representing a better status on insights and lucidity. However, we observed aggravated anxiety in patients with MHs than those with single pRBD. Previous studies have also found that patients with MHs experienced more anxiety than depression (Zhong et al., 2021). In our sample, age, disease duration, levodopa use, dopamine receptor agonists use, NMS-Quest scores, HAMA scores, HAMD scores, and PDQ-39 scores were significantly associated with the presence of MHs in pRBD in univariate analysis. However, after excluding the confounding factors of age and disease duration, only age, disease duration, NMS-Quest scores, and HAMA scores were included in the final prediction model. We found that longer disease duration and higher NMS-Quest scores were independent predictors of MHs in pRBD patients. However, as RBD and MHs may worsen with the progression of PD, the screening and treatment of anxiety should be the focus of care.

The present study has some limitations. First, this study was a cross-sectional study. RBD is an important clinical feature in the prodromal stage of PD. Although we found that most MHs occurred after the onset of motor symptoms in PD, we did not collect supplementary data on the temporal relationship between pRBD and PD, such as the onset of pRBD and MHs. Longitudinal studies are needed in the future to explore their occurrence and development. Second, the diagnosis of RBD in this study was made based on the RBDSQ questionnaire. Although we applied a clinically more stringent score of 6 as the cutoff value, there may still have been some false-positive RBD patients. As the gold standard for the diagnosis of RBD, polysomnography should be more widely used in future studies to increase the accuracy of the research. Third, the sample size of this study was relatively small after subgroup grouping and more large-sample, multicenter studies are needed in the future. Finally, although our study involved a relatively structured and standardized survey of minor hallucinations, it is still necessary to establish a unified assessment scale to assess the clinical characteristics of MHs comprehensively. Such a scale will provide a better reference for future research on minor hallucinations in different PD subgroups.

## 5. Conclusion

A high prevalence of MHs was observed in PD patients with pRBD. The main type of MHs in patients with pRBD was presence hallucinations, which may develop into VHs. This study provides evidence that MHs in PD with RBD are mainly associated with disease duration and severity of nonmotor symptoms. More neuroimaging studies and longitudinal observations focused on MHs in RBD subtypes of PD are necessary further to investigate the underlying mechanism and evolvement of MHs.

## Data availability statement

The original contributions presented in the study are included in the article/supplementary material, further inquiries can be directed to the corresponding author.

## Ethics statement

The study was approved by the Ethics Committee of the Affiliated Brain Hospital of Nanjing Medical University (grant numbers 2021-KY007-01). All procedures followed the Declaration of Helsinki.

## Author contributions

YJ, LZ, and FH conceived and designed the study. LZ, YP, JY, and XJ obtained the funding. YJ, YW, YC, DL, JZ, XJ, and BS collected the data. YJ, JZ, and YZ conducted the data analysis. YJ drafted the manuscript. All authors contributed to the article and approved the submitted version.

## Funding

This research was funded by the National Natural Science Foundation of China (grant numbers: 82171249 and 82101332), the Jiangsu Provincial Cadre Health Projects (grant number: BJ20005), the Special Funds of the Jiangsu Provincial Key Research and Development Program (grant number: BE2019612), the Jiangsu Province Elderly Health Project (grant numbers: LD2021013 and LR2021018), the Nanjing Medical University School Fund Project (grant number: NMUB20210223), the Nanjing Medical Science and Technology Development Foundation (grant number: QRX17026), the Nanjing Rehabilitation Medicine Center Project, and the Nanjing Industrial and Information Technology Development Special Fund Project.

## Conflict of interest

The authors declare that the research was conducted in the absence of any commercial or financial relationships that could be construed as a potential conflict of interest.

## Publisher’s note

All claims expressed in this article are solely those of the authors and do not necessarily represent those of their affiliated organizations, or those of the publisher, the editors and the reviewers. Any product that may be evaluated in this article, or claim that may be made by its manufacturer, is not guaranteed or endorsed by the publisher.
